# Dynamic Changes in Cell Wall Polysaccharides during Fruit Development and Ripening of Two Contrasting Loquat Cultivars and Associated Molecular Mechanisms

**DOI:** 10.3390/foods12020309

**Published:** 2023-01-09

**Authors:** Honghong Deng, Xi Wang, Yang Wang, Yinchun Xiang, Mingmin Chen, Huifen Zhang, Xian Luo, Hui Xia, Dong Liang, Xiulan Lv, Jin Wang, Qunxian Deng

**Affiliations:** College of Horticulture, Sichuan Agricultural University, Chengdu 611130, China

**Keywords:** loquat, fruit firmness, cell wall polysaccharides, enzyme activity, gene expression

## Abstract

Loquats have drawn much attention due to their essential nutrients and unusual phenology, which fills a market gap in early spring. Fruit firmness (FF) is one of the most important quality attributes. Dynamic changes in FF, cell wall (CW) polysaccharides, CW hydrolase activity, and expression of CW metabolism-related genes during the fruit development and ripening stages of two contrasting loquat cultivars were compared. Although the two cultivars possessed similar FF at the initial fruitlet stage, Dawuxing was significantly firmer than Ninghaibai at all subsequent time points. FF was positively correlated with the contents of covalent-soluble pectin and hemicellulose, activity of peroxidase, and gene expressions of *PME*, *EG*, *CAD*6, and *POD*; and negatively correlated with the contents of water-soluble pectin, activities of polygalacturonase, endo-glucanase, cellobiohydrolase, and xylanase, and gene expressions of *PG*, *EG*2, *PAL*1, *PAL*3, and *CAD*5. Identifying molecular mechanisms underlying the differences in FF is useful for fundamental research and crop improvement in future.

## 1. Introduction

Loquat (*Eriobotrya japonica* Lindl.), taxonomically belonging to the subfamily Maloideae of the family Rosaceae, is a commercially and nutritionally important subtropical evergreen fruit crop [1,2]. Native to Southwestern and South-central China, it has been extensively cultivated in more than 30 countries, including China, Spain, Japan, Turkey, Korea, Tukey, India, Pakistan, South Africa, and the United States [1]. As consumers become significantly health-conscious, loquat has attracted special attention because of its abundance in health-promoting compounds, such as vitamins, minerals, phenolics, carotenoids, flavonoids, and triterpenic acids [3,4]. These compounds are also associated with antioxidant and free radical-scavenging activities [2,5,6].

In contrast to other temperate fruit crops, the loquat trees bloom in autumn and early winter and its fruit matures in spring and early summer, a time when very few fleshy fruits are available in the fresh marketplaces [2]. This unusual phenology makes it a popular fruit crop for consumers and a profitable crop for growers. Both fresh and processed loquat fruits are consumed worldwide and their consumption and production have increased rapidly.

Fruit texture is one of four important quality attributes of fresh fruit, along with appearance, flavor, and nutritional properties. Fruit texture is often described by firmness, crispness, hardness, mealiness, flouriness, and grittiness. Firmness is the primary measure of fresh fruit texture [7] in determining consumer satisfaction and appreciation [8], post-harvest storability and shell-life performance [9], and packaging and transportation methods [10]. Firmness change is the most noticeable textural change that occurs during fruit development and ripening [11], and it is usually quantified using an instrumental texture profile analysis [12]. This important textural feature depends, at least in part, on the structural modification, disassembly, and intercellular adhesion reduction of the primary cell wall (CW) polysaccharide [11]. Plant CW mainly consists of complex networks of polysaccharides (pectin, hemicellulose, cellulose, and lignin) and structural proteins [13,14].

As a coordinated event, polysaccharide modification or disassembly requires orchestrated participation of a wide variety of enzymes. For example, polygalacturonase (PG) plays a crucial role in pectin disassembly [15], whereas pectinesterase (PE), also called pectin methylesterase (PME), is involved in both remodeling and pectin disassembly [16]. Three types of cellulases, namely endo-glucanase (EG), cellobiohydrolase (CBH), and β-glucosidase (BG), act synergistically in cellulose decomposition [17]. Xylanases (Xyls) are a class of enzymes involved in the depolymerization of linear polysaccharides into simple monosaccharides and xylooligosaccharides, such as beta-1,4-xylan into xylose, thus breaking down hemicellulose [18]. Phenylalanine ammonia lyase (PAL), cinnamyl alcohol dehydrogenase (CAD), and peroxidase (POD) play pivotal roles in the lignin biosynthesis pathway [19].

To understand the textural changes of fruit, the compositional and architectural changes of the CW and its interaction with numerous enzymes that cause its degradation is an important task that needs to be elucidated. Although a few studies have reported on the textural changes in loquats during post-harvest storage [19,20,21], knowledge regarding textural changes during fruit development and ripening remains limited.

Therefore, in the present study, we evaluated fruit firmness and the CW compositions and content during seven representative stages of fruit development and ripening in two contrasting loquat cultivars. The corresponding changes in gene expression and enzyme activity concomitant with fruit development and ripening were also determined. The outcomes of this study will reveal the physiological processes and molecular mechanisms underlying textural changes in loquat fruit. The comparisons between the contrasting cultivars will provide an excellent opportunity to identify the factors affecting loquat texture.

## 2. Materials and Methods

### 2.1. Plant Materials

The red-fleshed ‘Dawuxing’ (DWX, *E. japonica* Lindl.) and white-fleshed ‘Ninghaibai’ (NHB, *E. japonica* Lindl.), categorized as firm and soft loquat cultivars, respectively, were used in this experiment. The trees were seven years old (in 2020) grown in a commercial loquat orchard located in Longquanyi District, Chengdu City, Sichuan Province, China (30°62′24″ N, 104°32′18″ E) with identical horticultural practices.

**Figure 1:**
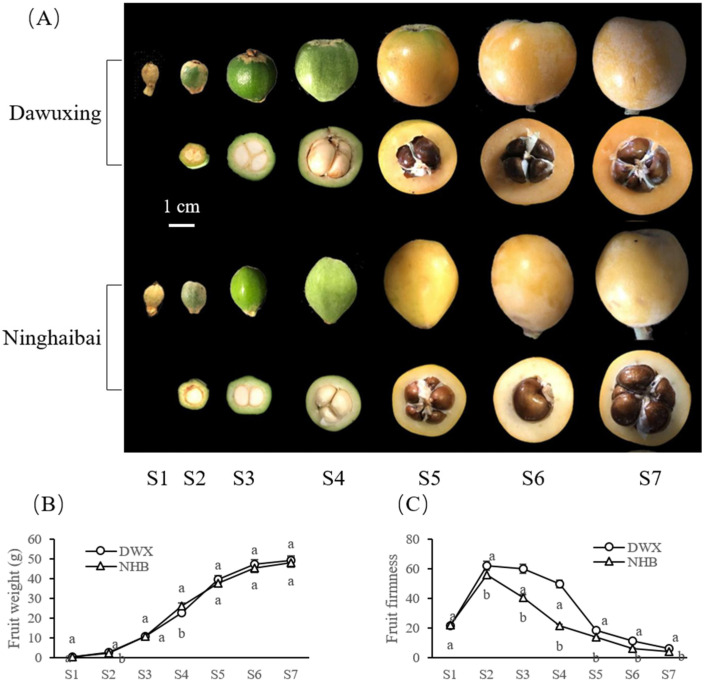
Fruit phenotype (**A**), weight (**B**), and firmness (**C**) changes during fruit development and ripening of the Dawuxing and Ninghaibai loquat. The x axis represents seven different developmental stages of loquat fruit: 50, 110, 130, 150, 160, 170, and 180 days after full bloom (DAFB). The transverse sections of fruits corresponding to these developmental stages are shown in (**A**). Scale bar represents 1 cm. Values are means ± SD of three biological replicates. Variance analysis was carried out independently for each of the seven developmental stages studied. The different lowercase letters in the line charts indicate significant differences (*p* < 0.05).

The seven representative stages (Figure 1A) of loquat fruit development and ripening are: young fruit (fruitlet) (S1), stunted growth of fruitlet (S2), cell division (immature green) (S3), cell expansion (mature green) (S4), breaker (veraison) (S5), half-ripe (S6), and full-ripe (S7), which correspond to 50, 110, 130, 150, 160, 170, and 180 days after full bloom (DAFB), respectively. Loquat fruit growth phases were established based on Fu et al.’s [22] and our previous observations. The time point when the entire inflorescence had approximately 75% of its flowers open at the full-bloom stage was set as the baseline (0 DAFB). Loquat fruits grow rapidly approximately one month before harvest and were thus sampled at 10-day intervals from 150 to 180 DAFB. Thirty fruits were randomly collected from the upper, middle, and lower canopy of each tree. The fruit samples from five trees consisted of one biological replicate. Each stage consisted of three biological replicates.

### 2.2. Determination of Fruit Firmness and Weight

Fruit firmness was determined in two opposite directions in the equatorial zone of each fruit using a TMS-Pilot precision texture analyzer (TL-Pro testing system, FTC, Atlanta, GA, USA) with a P/5 probe (5 mm diameter). Each measurement was made using the pre-, test-, and post-speed parameters of 1.0 mm s^−1^ and a depth of 5 mm in 5.0 s with two technique replicates. Fruit weight was determined using a 0.01 g sensitive balance and obtained from an average of 10 fruits in each biological replicate.

### 2.3. Extraction and Determination of Cell Wall Components

CW constituents, including pectin, cellulose, and hemicellulose, were extracted and determined according to the methods described previously by Chen et al. [23] and Huang et al. [24] with minor modifications. Briefly, 3.0 g of frozen fruit samples were grounded into a fine powder in liquid nitrogen and homogenized in 80% ethanol before boiling for 20 min. After rapid cooling, the homogenate extracts were centrifuged at 12,000 g at 4 °C for 20 min. The pellet was sequentially washed with 10 mL 80% ethanol and 10 mL pure acetone. The final insoluble material was then dried overnight at 45 °C.

Different solvents were sequentially used to separate pectin fractions (water-soluble pectin (WSP), ionic (EDTA)-soluble pectin (ISP), covalent (Na_2_CO_3_)-soluble pectin (CSP)), hemicellulose (KOH-soluble), and cellulose (H_2_SO_4_-soluble) [23]. Different forms of pectin were determined using the carbazole–sulfuric acid method, in which galacturonic acid was used to create a standard curve, as described previously by Chen et al. [23]. Hemicellulose content was determined using the anthrone colorimetric method [23]. Cellulose content was determined using a CLL-2-Y assay kit (Suzhou Keming Biotechnology Co. Ltd., Suzhou, China) following the manufacturer’s instructions. Lignin was extracted and quantified using the method of Cai et al. [19].

### 2.4. Extraction of Cell-Wall-Degrading Enzymes and Determination of Activity

PG, PME, SOD, and POD activities and MDA content were analyzed using the available commercial PG-2-G, PME-2-G, SOD-2-Y, POD-2-Y, and MDA-2-Y assay kits (Suzhou Keming Biotechnology Co., Ltd., Suzhou, China), respectively, following the manufacturer’s instructions. Cellulases were extracted and their activities were determined following the methods described by Cai et al. [19].

### 2.5. Quantitative Real-Time Polymerase Chain Reaction (qRT-PCR)

Total RNA from the fruit samples was extracted using TRIzol^®^ reagent (Invitrogen, Carlsbad, CA, USA) and treated with a TURBO DNA-free^™^ kit (Ambion, Austin, TX, USA) to remove DNA contamination following the manufacturers’ protocol. Purified RNA was quantified using a Nanodrop 2000 spectrophotometer (Thermo Fisher Scientific, Carlsbad, Inc., Carlsbad, CA, USA) and RNA integrity was evaluated using 1% agarose gel electrophoresis. First-strand complementary DNA synthesis was conducted using a reverse transcription kit containing a PrimeScript^TM^ RT reagent kit and gDNA Eraser (Perfect Real Time) (Takara, Dalian, China).

The detailed sequences of primers used are listed in Table 1; moreover, qRT-PCR was performed on a CFX96 Touch Real-Time PCR C1000 Thermal Cycler system (Bio-Rad, Hercules, CA, USA) using Brilliant III Ultra-Fast SYBR Green QPCR Master Mix (Agilent Technologies Inc., Santa Clara, CA, USA) following the manufacturer’s instructions. The internal reference genes for the normalization of relative gene expression were obtained from Fu et al. [22]. Three technical replicates were performed for each biological replicate.

### 2.6. Statistical Analyses

Means and standard deviations (SD) were calculated using Microsoft Excel software. Differences within cultivars at each time point were statistically evaluated by a one-way analysis of variance, followed by comparisons using Tukey’s honest significant difference (HSD) test with a statistical significance of *p* < 0.05. The association between CW components and other continuous variables (CW degradation enzyme activities and associated gene expressions) was determined using Pearson’s correlation coefficient (*p* < 0.05). For qRT-PCR, the 2^−ΔΔCT^ method was used to calculate gene expression levels after normalization to the internal reference gene.

## 3. Results

### 3.1. Comparative Analyses of Fruit Phenotype, Weight, and Firmness Changes of Two Contrasting Loquat Cultivars

The different external phenotypes and transverse sections during fruit development and ripening of DWX and NHB loquats are shown in Figure 1A. The development of DWX and NHB fruits encompassed an average period of approximately 180 d. The fruit weight curves of DWX and NHB loquats followed a single-sigmoid pattern, with an extremely slow growth in the fruitlet stages (S1–2) and a dramatic increase during the expansion stages (S3–5), followed by a gradual increase (S5–7). At harvest, no significant differences (*p* > 0.05) were observed between the fruit weight of DWX (49.33 ± 0.47 g) and NHB (48.33 ± 0.47 g) (Figure 1B). Fruit firmness exhibited a steep increase from the young fruitlet stage (S1) to the stunted growth stage of fruitlet (S2), and then softened in later stages of fruit development and ripening (S3–7). The fruit firmness of DWX was significantly higher (*p* < 0.05) than that of NHB at all time points except for the fruitlet stage (S1), with the DWX being 1.51-times higher than the NHB at harvest (S7) (Figure 1C).

### 3.2. Comparative Analyses of Cell Wall Compositions of Two Contrasting Loquat Cultivars

The WSP and ISP contents were relatively low initially and gradually increased until the breaker stage (S5), after which a rapid increase occurred (S5–7). The WSP contents during the breaker and ripening stages (S5–7) of DWX loquat (12.90~25.87 mg g^−1^ FW) were significantly lower (*p* < 0.05) than those of NHB loquat (18.17~38.93 mg g^−1^ FW); however, the ISP contents for the DWX loquat (20.97~32.47 mg g^−1^ FW) were significantly higher (*p* < 0.05) than those of NHB loquat (15.94~29.60 mg g^−1^ FW).

The hemicellulose content of DWX and NHB loquats peaked during the early developmental stage (S1) with maximum values of 3.53 ± 0.20a and 2.25 ± 0.07b mg g^−1^ FW, respectively. Subsequently, a gradual decline was observed during the fruit cell expansion stages (S3–5), followed by a slight increase in the breaker and ripe stages (S5–7). At harvest, the hemicellulose content was significantly higher (*p* < 0.05) in DWX loquat (1.57 ± 0.04 mg g^−1^ FW) relative to NHB loquat (1.37 ± 0.06 mg g^−1^ FW).

The CSP and cellulose contents gradually increased, peaking at the cell expansion stages (S3–4), however, they decreased thereafter. The CSP and cellulose contents of DWX loquat during the entire period were significantly higher (*p* < 0.05) than those of NHB loquat.

**Figure 2:**
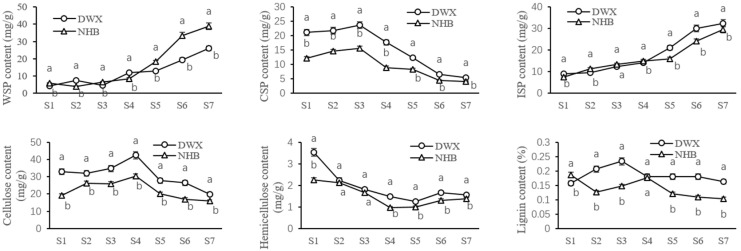
Dynamic changes of water-soluble pectin (WSP), ionic-soluble pectin (ISP), covalent-soluble pectin (CSP), cellulose, hemicellulose, and lignin contents at seven different stages during loquat fruit development and ripening. The stages numbered on the *x*-axis correspond to those presented in Figure 1. Values are means ± SD of three biological replicates. Variance analysis was carried out independently for each of the seven developmental stages studied. The different lowercase letters in the line charts indicate significant differences (*p* < 0.05).

The lignin content of the DWX loquat increased during the early developmental stages (S1–3), rapidly decreased after the cell division stage (S3–5) and remained relatively stable over the breaker and ripe stages (S5–7). An opposite trend was observed for the NHB loquat, whereby the entire lignin content initially decreased (S1–2), followed by a substantial increase from the cell division stages (S3–5). The lignin content was significantly higher (*p* < 0.05) in DWX loquat (0.16–0.18 mg g^−1^ FW) than in NHB loquat (0.10~0.12 mg g^−1^ FW) during the breaker and ripe stages (S5–7) (Figure 2).

### 3.3. Comparative Analyses of Enzyme Activities for Cell Wall Metabolism of Two Contrasting Loquat Cultivars

**Figure 3:**
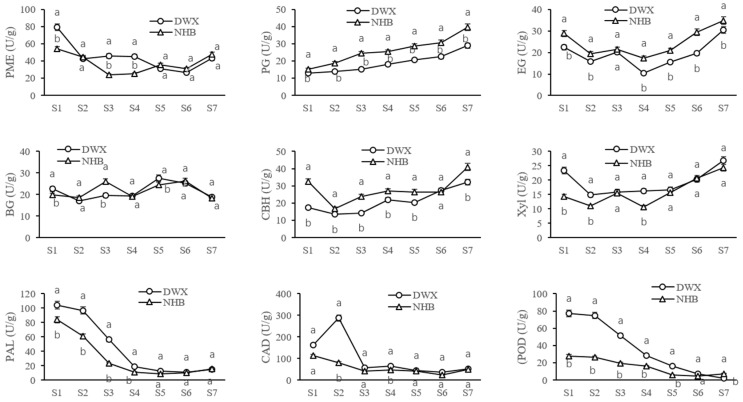
Dynamic changes of pectin methylesterase (PME), polygalacturonase (PG), endo-glucanase (EG), β-glucosidase (BG), cellobiohydrolases (CBH), xylanase (Xyl), phenylalanine ammonia lyase (PAL), cinnamyl alcohol dehydrogenase (CAD), and peroxidase (POD) enzyme activities at seven different stages during loquat fruit development and ripening. The stages numbered on the *x*-axis correspond to those presented in Figure 1. The enzymes activities were expressed as units of activity per g fresh weight. Values are means ± SD of three biological replicates. Variance analysis was carried out independently for each of the seven developmental stages studied. The different lowercase letters in the line charts indicate significant differences (*p* < 0.05).

The activities of key CW metabolism-related enzymes in DWX and NHB loquats during development and ripening were measured (Figure 3). The PME activity initially decreased in the early developmental stage and tended to be stable over time, followed by a rapid increase at maturity. The PME activity was significantly higher (*p* < 0.05) in NHB loquat (30.94–47.75 U g^−1^ FW) than in DWX loquat (26.59–43.18 U g^−1^ FW) during the breaker and ripe stages. Furthermore, PG activity progressively increased during the entire period, and the rate of increase significantly accelerated during the ripening stages. PG activity was significantly higher (*p* < 0.05) in the NHB loquats throughout all developmental stages than in DWX loquats, which was in contrast to the CSP accumulation pattern.

The EG and CBH activities exhibited similar initial decreasing trends. A weak peak or shoulder of EG enzyme activity was discernible at the early fruit expansion stage, when a gradual increase was observed for CBH enzyme activity; thereafter, both EG and CBH enzyme activities rapidly increased as fruit ripening progressed. Compared to DWX loquat, the EG and CBH enzyme activities of NHB loquat were significantly higher (*p* < 0.05) throughout all developmental stages. BG enzyme activity fluctuated with two peaks detected at the early expanding and later developmental stages.

The changes in Xyl enzyme activity during loquat fruit development and ripening were similar to the changes observed in EG and CBH enzyme activities, with a slight difference between different cultivars at the fruit expansion stages. When the activity of Xyl in DWX remained stable, it rapidly increased and then declined in NHB. Significantly higher (*p* < 0.05) Xyl activity was observed in DWX loquat than in NHB loquat.

PAL, CAD, and POD activities in the two loquat cultivars exhibited similar changing patterns. They significantly decreased from the fruitlet to expansion stages and then remained at a relatively low level or experienced very minor changes from the breaker–ripe transition stage. The only exception was a sharp and short-term spike in the stunted growth stage (S2) in the DWX fruitlet. Compared to NHB loquat, DWX loquat maintained significantly higher (*p* < 0.05) PAL, CAD, and POD activities before the breaker stage, however, the differences were not significant (*p* > 0.05) subsequently (Figure 3).

### 3.4. Correlation Analysis of Cell Wall Components and the Cell-Wall-Degrading Enzyme Activities of Two Contrasting Loquat Cultivars

The correlations between CW components and metabolism-related enzymatic activities are summarized in Table 2. A *p*-value representing the probability in the Tukey’s HSD test was used to compare the confidence coefficient among the data sets. Significant correlation between data sets is indicated if the *p*-value was less than 0.05 (*p* < 0.05, *) or 0.01 (*p* < 0.01, **). Strong significant negative correlations were apparent between loquat fruit firmness and the WSP content (r = −0.710 **) and enzymatic activities of CBH (r = −0.764 **), PG (r = −0.672 **), EG (r = −0.618 *), and Xyl (r = −0.603 *), whereas the fruit firmness was significantly positively correlated with the content of CSP (r = 0.841 **) and hemicellulose (r = 0.628 *) and POD activity (r = 0.625 *).

WSP content was significantly positively correlated with PG activity (r = 0.876 **), whereas CSP content was significantly negatively correlated with PG activity (r = −0.840 **). The negative correlations between hemicellulose content and Xyl enzyme activity (r = −0.218) and between cellulose content and the activities of CBH (r = −0.398) and BG (r = −0.207) were not significant (*p* > 0.05). Unexpectedly, we identified a strong negative correlation between hemicellulose content and EG enzyme activity (r = −0.712 **).

The positive correlation coefficients between lignin and the activities of PAL, CAD, and POD were 0.382, 0.377, and 0.525, respectively, although they were not significant (*p* > 0.05).

### 3.5. Comparative Analyses of Gene Expressions for Cell Wall Metabolism of Two Contrasting Loquat Cultivars

**Figure 4:**
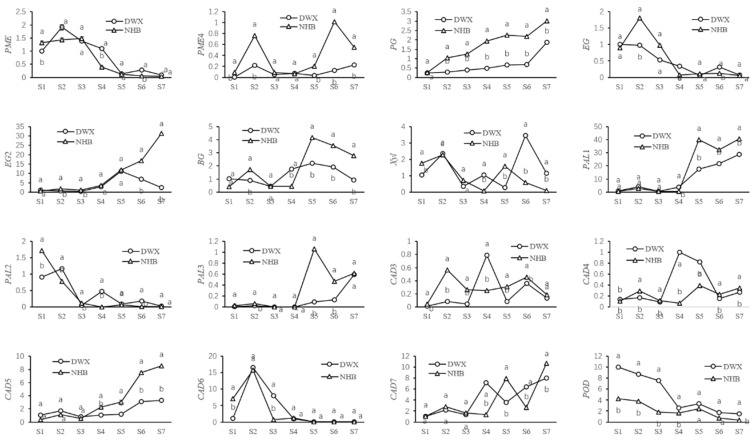
Dynamic changes of pectin methylesterase (*PME*), polygalacturonase (*PG*), endoglucanase (*EG*), β-glucosidase (*BG*), cellobiohydrolases (*CBH*), xylanase (*Xyl*), phenylalanine ammonia lyase (*PAL*), cinnamyl alcohol dehydrogenase (*CAD*), and peroxidase (*POD*) gene expressions at seven different stages of loquat fruit development and ripening. The stages numbered on the *x*-axis correspond to those presented in Figure 1. Values are means ± SD of three biological replicates. Variance analysis was carried out independently for each of the seven developmental stages studied. The different lowercase letters in the line charts indicate significant differences (*p* < 0.05).

The expression of key CW metabolism-related genes in the two loquat cultivars during the development and ripening stages was analyzed. All gene expression analyses were normalized to internal reference genes. Overall, the gene expression profiles were developmentally regulated and the two loquat cultivars exhibited distinct gene expression profiles throughout the fruit developmental stages (Figure 4).

DWX and NHB exhibited high expression of *PME* at the fruitlet and expansion stages (S1–4) and expression decreased as the fruit matured (S5–7). In general, the *PME*4 expression profiles of the two loquat cultivars changed in a similar manner as they peaked at the stunted growth stage of the fruitlet, declined to a very low level until the breaker stage (S5), and finally increased during the subsequent fruit ripening stages. A significantly higher (*p* < 0.05) expression of *PME*4 was observed across almost all stages in NHB loquats compared to a low expression of *PME*4 in the DWX loquat. *PG* exhibited a consistently increasing trend during fruit development, with this pattern being more evident during fruit maturation. When comparing the *PG* expression of the two loquat cultivars, NHB recorded significantly higher (*p* < 0.05) values throughout all stages (except the fruitlet stage).

The expression profiles of *EG* in the two cultivars demonstrated an overall downward trend. However, the expression in NHB loquat reached a large peak at the stunted growth stage of fruitlet (S2) and DWX reached a weak peak (S6) before slowly decreasing until fruit maturation. The *EG*2 gene initially remained low with a stable expression and gradually increased as the fruit expanded. However, its expression began to dramatically increase in NHB loquats and sharply decrease in DWX from the breaker stage. The *BG* gene in DWX gradually decreased, increased, and then decreased again, whereas the *BG* gene in NHB initially decreased slowly, and then increased dramatically. Fluctuating expressions of *Xyl* were identified in the two cultivars and the highest expression levels were found at S6 in DWX and S2 in NHB.

The *PAL*1 and *PAL*3 genes showed a basal expression pattern in the fruitlet and fruit expansion stages, followed by a sustained increase, thereby presenting the highest expression levels at the maturation stage. Specifically, a unique peak in expression at the breaker stage of the NHB loquat was observed. The *PAL*2 gene in the NHB loquat cultivar exhibited an initial peak followed by an abrupt decline and a weak peak at S6. The *PAL*2 showed an expression peak in DWX loquat cultivar at the stunted growth of fruitlet (S2). In addition, a second peak expression of *PAL*2 was recorded in the DWX loquat cultivar at the half-ripe stage (S6).

The *CAD*3 gene in DWX maintained a low expression level before the cell division stage, when it was evidently upregulated in NHB loquat. The expression of *CAD*3 in DWX peaked at the cell expansion stage, whereas it remained steady in NHB before increasing again at maturation. *CAD*4 was stably expressed at low levels in DWX loquat; however, the *CAD*4 gene expression level was significantly (*p* < 0.05) higher in NHB than in DWX and was highest at the cell expansion and breaker stages. The *CAD*5 gene showed slightly increasing expression changes from the early fruitlet to the cell expansion stage, increasing quickly at later stages and peaking at maturation. *CAD*5 gene expression was clearly higher in NHB than in DWX at all stages examined. *CAD*6 gene expression increased sharply at the stunted growth stage of fruitlets before subsequently declining rapidly to a low level until the breaker stage. A fluctuating expression of *CAD*7 was recorded in both DWX and NHB loquats, reaching maximum values at the ripe stage. The *POD* gene exhibited a declining trend throughout development and maturity and remained significantly higher (*p* < 0.05) in DWX than in NHB throughout all developmental stages.

### 3.6. Correlation Analysis of Cell Wall Components, Cell-Wall-Degrading Enzyme Activities, and Gene Expressions of Two Contrasting Loquat Cultivars

The correlations among CW components, CW-degrading enzyme activities, and gene expression are summarized in Table 3. We observed significant positive correlations between loquat fruit firmness and the gene expressions of *PME* (r = 0.884 **), *CAD*6 (r = 0.756 **), and *POD* (r = 0.552 *), and negative correlations between loquat fruit firmness and the gene expressions of *PG* (r = −0.600 *) and *EG*2 (r = −0.596 *). For pectin, there were strong positive correlations between the gene expression of *PME* and CSP content (r = 0.836 **), gene expression of *PG* and WSP content (r = 0.778 **), and activity of PG enzyme (r = 0.959 **). We also observed negative correlations between the gene expression of *PME* and WSP content (r = −0.766 **) and between gene expression of *PG* and ISP content (r = −0.778 **).

**Figure 5:**
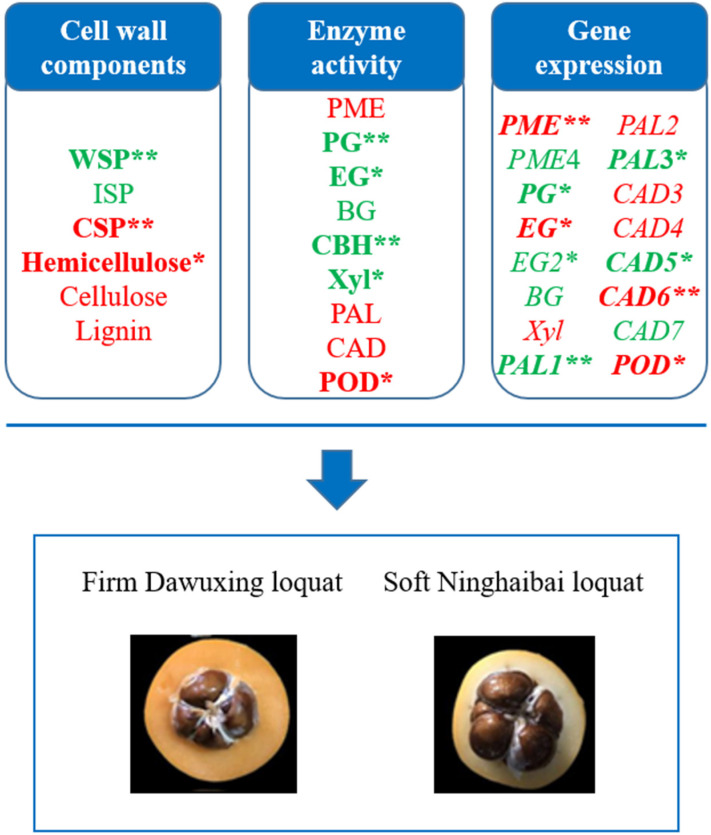
Schematic summary of the important factors influencing loquat fruit firmness. The bold represents statistically significantly related to loquat fruit firmness. The red and green represent positively and negatively correlated factors to loquat fruit firmness. * denotes *p* < 0.05; ** denotes *p* < 0.01.

The correlation coefficients of the gene expression of *EG*2 and the cellulose content and enzyme activity of EG were r = −0.45 and r = 0.52 (*p* > 0.05), respectively. The lignin content was significantly positively correlated with *POD* gene expression (r = 0.551 *). The gene expression levels of *PAL*2, *CAD*6, and *POD* were significantly positively correlated with the activities of PAL (r = 0.834 **), CAD (r = 0.663 **), and POD (r = 0.961 **), respectively. A schematic diagram summarizing the important factors influencing loquat fruit firmness are shown in Figure 5.

## 4. Discussion

### 4.1. Flesh Firmness Changes during Loquat Fruit Development and Ripening

The high economic and nutritional properties of loquat and its unusual phenology have grasped the attention of global consumers, growers, and researchers. Fruit firmness is among the most important quality attributes and is a hallmark of fruit ripening in fleshy fruits [7]. Loquat firmness is a controversial subject and varies considerably among cultivars and among post-harvest treatments [5,19,24]. Although the changes in fruit firmness exhibited in loquat fruits after harvest have been well characterized [5,19,24], very little information is currently available regarding the changes during the fruit development and ripening stages. In addition, pre-harvest fruit quality is a prerequisite for post-harvest fruit quality. Therefore, it is crucial to extend the dynamic changes in loquat fruit firmness during the developmental and ripening stages.

Loquat fruits are generally sorted into red- and white-fleshed cultivars [25]. Although the red-fleshed DWX and white-fleshed NHB loquats are categorized as the common loquat (*E. japonica* Lindl.), they exhibited contrasting fruit firmness. Similar botanical origins but contrasting fruit firmness between the two loquat cultivars provide an excellent opportunity to elucidate the mechanism underlying loquat fruit firmness. Therefore, the differences in fruit firmness-related factors between the two contrasting loquat cultivars were comprehensively compared. Loquat fruit firmness changed continuously during the development and ripening stages, showing an initial steep increase followed by a prominent progressive reduction. Although the two loquat cultivars had similar firmness values at the initial fruitlet stage, DWX loquat was significantly firmer than NHB loquat at all subsequent time points (Figure 1C).

### 4.2. Pectin Metabolism in Flesh Firmness Changes during Loquat Fruit Development and Ripening

In dicot plants, pectin contributes to approximately one-third of CW and is widely associated with fruit firmness [9]. Fruit firmness decreased as the loquat fruit grew and ripened, which was accompanied by an increase in WSP and ISP contents and a decline in CSP content (Figure 2, Table 2). These results are supported by previous research demonstrating that fruit ripening is involved in an increase in the solubility of pectin polysaccharides (WSP and ISP) and facilitates a decreasing trend of chelate-soluble pectin (CSP) [9]. As previously proposed, a reduction in CSP content and the simultaneous accumulation of WSP during fruit development strongly suggested that water-insoluble pectins were converted into water-soluble pectins [7], such as it was observed in strawberry [26], blueberry [27], Chinese bayberry [28], and watermelon [29]. In this study, the soft flesh cultivar (NHB loquat) had a significantly higher WSP content than the firm flesh cultivar (Figure 2). Loquat fruit firmness was significantly positively correlated with the CSP content and positively correlated with the WSP content (Table 2), demonstrating that loquat fruit firmness was closely correlated to the catabolism of covalently bound pectin.

The displacement of CSP to WSP indicates that structural modifications occurred during loquat fruit development and ripening. PME is an important enzyme involved in the fruit ripening period that catalyzes the demethylesterification of homogalacturonans and mediates pectin reconstruction [30]. Although PME enzyme activity was not significantly correlated with loquat firmness in the present study (Table 2), this enzyme could be associated with other CW modifications, such as decreasing methyl esterification, thereby enabling an increase in the substrate availability to other CW hydrolases [31]. A significantly higher PME enzyme activity in NHB loquat than in DWX loquat in the later developmental stages (Figure 3) coincided with the WSP accumulation pattern shown in Figure 2.

PG is a hydrolytic enzyme that depolymerizes homogalacturonans and is essential for softening and promoting textural changes in fleshy fruits, especially during late ripening [32]. In this study, PG activity was positively correlated with WSP content and negatively correlated with CSP content (Table 2). In addition, PG activity was significantly higher in NHB loquat than in DWX loquat throughout all developmental stages, which contrasted with the CSP accumulation pattern. Overall, the PG enzyme exhibited a significant effect on pectin content in loquat.

### 4.3. Cellulose and Hemicellulose Metabolism in Flesh Firmness Changes during Loquat Fruit Development and Ripening

Cellulose, the most abundant plant CW polysaccharide, is composed of (1 → 4)-β-glucan units [14]. Cellulases, which are represented by three key enzymatic activities (EG, CBH, and BG) are widely regarded as the most effective enzymes for converting cellulose to glucose [17]. In this study, although the negative correlation between flesh firmness and cellulose content was not significant (Table 2), cellulose content in the firm DWX loquat during the entire period was significantly higher than that of the soft NHB loquat (Figure 2). Celluloses are highly stable structures that play an important role in fruit firmness [33,34]. Loquat fruit firmness was negatively correlated with the enzyme activities of CBH, EG, and BG (Table 2), and the gene expression of *EG*2 (Table 3).

In the CW structure, cellulose and hemicellulose are embedded in a matrix of pectins [14], the latter being the most complex component of the CW [34]. In this study, the CW composition of both loquat cultivars showed a continuous decrease in the hemicellulose content (Figure 2). This situation corresponds with that of other species, such as apple (*Malus domestica* Borkh.) [35], pear (*Pyrus pyrifolia Nakai*) [36], melon (*Cucumis melo* L.) [37], sweet cherry (*Prunus avium*), and blueberry (*Vaccinium corymbosum* L.) [38]. In addition, owing to the significantly higher amount of hemicellulose in the DWX loquat than in the NHB loquat (Figure 2), a higher hardness was observed in the DWX loquat fruit (Figure 1), and the fruit firmness was significantly positively correlated with hemicellulose content (Table 3).

### 4.4. Lignin Metabolism in Flesh Firmness Changes during Loquat Fruit Development and Ripening

After cellulose, lignin is the most abundant polyphenolic polymer and contributes greatly to the CW structural support [39]. Lignification, caused by lignin accumulation and biosynthesis, is typically expressed as an increase in the firmness of loquat flesh [19,40]. The flesh lignification phenomenon of loquat has been thoroughly investigated during post-harvest storage, especially in low-temperature storage, which involves the coordinated action of a series of enzymes in the phenylpropanoid pathway, such as PAL, CAD, and POD [19]. The DWX loquat maintained significantly higher PAL, CAD, and POD enzyme activities before the breaker stage than NHB loquat; however, the differences were insignificant afterward (Figure 3). In agreement with our findings, Shan et al. [21] reported a close correlation between lignin contents among different loquat cultivars. In the current study, loquat fruit firmness was significantly negatively correlated with the expressions of *PAL*1, *PAL*3, and *CAD*5, but positively correlated with the expressions of *CAD*6 and *POD* (Table 3).

## 5. Conclusions

Fruit firmness is one of the most important quality attributes that determines consumer satisfaction and appreciation. This key feature depends largely on the structural modification or disassembly of the CW polysaccharide. In this study, the dynamic changes in fruit firmness, activity of CW hydrolase, and relative expression levels of CW metabolism-related genes of two contrasting loquat cultivars were compared. We identified changes in the molecular mechanisms underlying fruit firmness of different loquat cultivars during their fruit development and ripening, including pectin, cellulose, hemicellulose, and lignin metabolism. A better understanding of the composition, content, and accumulation of CW polysaccharide is necessary before genetic improvement or cultural practices can be made to loquat fruit texture. The comparisons of the two contrasting loquat cultivars provide an excellent opportunity to identify the factors that affect loquat fruit firmness.

## Figures and Tables

**Table 1:** Primers used for quantitative real-time polymerase chain reaction in this study.

Gene	F (Sequence 5′–3′)	R (Sequence 5′–3′)
*Actin*	AATGGAACTGGAATGGTCAAGGC	TGCCAGATCTTCTCCATGTCATCCCA
*PME* *1*	ATACAGGCGGATATTATC	ATCAAGTTGGTCTTCTTC
*PME* *4*	CACTATAATCTCTGGAACTC	TTAGGTCTCTTGCTATGA
*PG*	GTAACAGGTGATAATGGAA	CAGTATGTCGGATGAATG
*EG* *1*	TTACATCTCGCTCCACAA	AGACATAATCCGCCTGAG
*EG* *2*	TGATGCCTATGACAACTT	TGCCAATATACCGAGAAG
*BG*	ATTACTACTCAGGAACTTATG	TAGGAACGCCATTATACT
*Xyl*	AACCTCTTCCTTGACAAC	ACATATCCTTCGCTTACAG
*PAL* *1*	AACCAAGATGTCAACTCA	CAACTAGGAATGTGGATGA
*PAL* *2*	TTTGCCTACATTGATGAC	CATTCTTCTCACTCTCAC
*PAL* *3*	CAGTGCTACATATCCTCTAA	CATCTCCTTCTCACCATT
*CAD3*	AGATTGCGACTATTGTAGAG	TTGTAATCGTGCCATCAG
*CAD4*	GAGTAGGAGATGTGGTAG	GTATTGCTCATTGTCTGT
*CAD5*	CTGATGAGTTCTTGGTTAG	CTGATGAGTTCTTGGTTAG
*CAD6*	GCAGCAAAACATAACATAACGGC	CTAGCAGCCAGTGTGTTTCCAA
*CAD7*	ACTCGAGAGGCTAGTGAAGAAAG	CTAGGCTAGGCGGGAACTTTTAG
*POD*	TCTTGTGCTGATATTCTT	TTAGTCCATCCAATCTTC

**Table 2:** Correlations between the cell wall components and the cell wall-degrading enzyme activities.

Index	Firmness	PME	PG	EG	CBH	BG	Xyl	PAL	CAD	POD
firmness	1.000	0.097	−0.672 **	−0.618 *	−0.764 **	−0.412	−0.603 *	0.500	0.473	0.625 *
WSP	−0.710 **	−0.220	0.876 **	-	-	-	-	-	-	-
CSP	0.841 **	0.438	−0.840 **	-	-	-	-	-	-	-
ISP	−0.380	−0.182	0.354	-	-	-	-	-	-	-
hemicellulose	0.628 *	-	-	−0.712 **	−0.531	−0.323	−0.218	-	-	-
cellulose	0.285	-	-	0.022	−0.398	−0.207	0.161	-	-	-
lignin	0.525	-	-	-	-	-	-	0.382	0.377	0.525

Note: “-” means that no correlation analysis has been performed. The asterisks indicate significant differences (*: *p* < 0.05; **: *p* < 0.01).

**Table 3:** Correlation analysis of cell wall components and hydrolase activities with gene expression.

Index	*PME*	*PME4*	*PG*	*EG*	*EG2*	*BG*	*XYL*	*PAL1*	*PAL2*	*PAL3*	*CAD3*	*CAD4*	*CAD5*	*CAD6*	*CAD7*	*POD*
firmness	0.884 **	−0.16	−0.600 *	0.647 *	−0.597*	−0.46	0.16	−0.720 **	0.35	−0.592 *	0.16	0.03	−0.619 *	0.756 **	−0.45	0.552 *
WSP	−0.766 **	0.549*	0.778 **	-	-	-	-	-	-	-	-	-	-	-	-	-
CSP	0.836 **	−0.45	−0.778 **	-	-	-	-	-	-	-	-	-	-	-	-	-
ISP	−0.573 *	−0.08	0.16	-	-	-	-	-	-	-	-	-	-	-	-	-
hemicellulose	-	-	-	0.14	−0.33	−0.37	−0.10	-	-	-	-	-	-	-	-	-
cellulose	-	-	-	0.677 **	−0.45	−0.43	0.30	-	-	-	-	-	-	-	-	-
lignin	-	-	-	-	-	-	-	−0.634 *	0.28	−0.633 *	−0.35	−0.06	−0.650 *	0.32	−0.44	0.551 *
PME	0.29	−0.13	−0.35	-	-	-	-	-	-	-	-	-	-	-	-	-
PG	−0.751 **	0.48	0.959 **	-	-	-	-	-	-	-	-	-	-	-	-	-
EG	-	-	-	−0.18	0.52	0.18	−0.19	-	-	-	-	-	-	-	-	-
CBH	-	-	-	−0.51	0.625 *	0.25	−0.18	-	-	-	-	-	-	-	-	-
BG	-	-	-	−0.27	0.18	0.42	−0.06	-	-	-	-	-	-	-	-	-
XYL	-	-	-	−0.42	0.42	0.23	−0.10	-	-	-	-	-	-	-	-	-
PAL	-	-	-	-	-	-	-	−0.600 *	0.834 **	−0.48	−0.43	−0.36	−0.46	0.676 **	−0.570 *	0.881 **
CAD	-	-	-	-	-	-	-	−0.41	0.715 **	−0.33	−0.35	−0.20	−0.30	0.663 **	−0.34	0.767 **
POD	-	-	-	-	-	-	-	−0.641 *	0.579 *	−0.550*	−0.37	−0.20	−0.49	0.556 *	−0.549 *	0.961 **

Note: A *p*-value that represents the probability in Tukey’s HSD test was used to compare the confidence coefficient among the data sets. Significant correlation between data sets is indicated if *p*-value is less than 0.05 (*p* < 0.05, *) or 0.01 (*p* < 0.01, **). “-” means that no correlation analysis has been performed.

## Data Availability

All data supporting the findings of this study are available within the paper.
